# Dental Examiners of Maryland. Validity of Their Appointment and Their Actions Questioned in a Suit Instituted

**Published:** 1898-01

**Authors:** 


### Editorial, Etc.

### DENTAL EXAMINERS OF MARYLAND. VALIDITY OF THEIR APPOINTMENT AND THEIR ACTIONS QUESTIONED IN A SUIT INSTITUTED.

The validity of the act of 1896 providing for the appointment of a State Board of dental examiners is questioned in a suit instituted in Circuit Court No. 2, yesterday by Will Gavitt Bradford, through A. S. J. Owens, attorney. Mr. Bradford applied for an injunction restraining the dental examiners from enforcing the act in question as far as he is concerned.

In his bill of complaint Mr. Bradford states that the degree of doctor of dental surgery was conferred upon him March 9, 1892, by the Ohio College of Dental Surgery, the second oldest dental college in the United States, and ranking second to none in this country. Subsequently, he states, certificates entitling him to practice his profession in Ohio and Kentucky were issued him by the dental examiners of those States, and he was also admitted to practise in Arkansas.

Mr. Bradford claims that his diploma entitles him to practice dentistry in Maryland. He arrived in Baltimore recently, he states, and was granted by the examiners a temporary certificate entitling him to practice dentistry from October 21 to November 11, 1897. On November 11 he was examined as to his qualifications, and he asserts that he knows he answered correctly 75 per cent. of the questions asked him, and that his mechanical and laboratory work was up to the required standard. Notwithstanding this, the bill states, he was refused a certificate, but the president and secretary of the board of examiners declined to inform him wherein he was deemed deficient.

He was told that he could not practice dentistry in Maryland, and that if he did so he would be arrested.

The act of Assembly under which the examiners were appointed is alleged to be unconstitutional, as it vests arbitrary and unlawful power in the board, whose action has deprived Mr. Bradford of a property right and privilege without due process of law. To state that Mr. Bradford is not qualified to practice dentistry, or his arrest for so practicing contrary to the provisions of the act, it is alleged, would do him irreparable injury. Even if the act is valid, the bill asserts, it does not apply to Mr. Bradford, as those holding certificates issued prior to its passage are excluded from its provisions.

The court is asked to enjoin the dental examiners from giving public or private notice that Mr. Bradford is not authorized to practice dentistry in Maryland, from enforcing the act in question against him and from causing his arrest if he does practice dentistry. An order signed by Judge Stockbridge requires cause to be shown by December 31, why the injunction should not be granted.

The board of examiners is composed of Drs. Edward P. Keech, President; J. C. Heuisler, Secretary; A. B. King, William T. Kelley, A. C. McCurdy and Edward Nelson. The penalty for violation of the act is a fine of from $50 to $300.—*Balto. Sun*, Dec. 23, '97.

